# Ultrastructural Characterization of the Glomerulopathy in Alport Mice by Helium Ion Scanning Microscopy (HIM)

**DOI:** 10.1038/s41598-017-12064-5

**Published:** 2017-09-15

**Authors:** Kenji Tsuji, Hani Suleiman, Jeffrey H. Miner, James M. Daley, Diane E. Capen, Teodor G. Păunescu, Hua A. Jenny Lu

**Affiliations:** 1 Center for Systems Biology, Program in Membrane Biology and Division of Nephrology, Department of Medicine, Massachusetts General Hospital, and Harvard Medical School, Boston, MA USA; 2 Department of Pathology and Immunology, Washington University School of Medicine, St. Louis, MO USA; 3 Division of Nephrology, Washington University School of Medicine, St. Louis, MO USA; 4 Research Laboratory of Electronics, Massachusetts Institute of Technology, Cambridge, MA USA

## Abstract

The glomerulus exercises its filtration barrier function by establishing a complex filtration apparatus consisting of podocyte foot processes, glomerular basement membrane and endothelial cells. Disruption of any component of the glomerular filtration barrier leads to glomerular dysfunction, frequently manifested as proteinuria. Ultrastructural studies of the glomerulus by transmission electron microscopy (TEM) and conventional scanning electron microscopy (SEM) have been routinely used to identify and classify various glomerular diseases. Here we report the application of newly developed helium ion scanning microscopy (HIM) to examine the glomerulopathy in a *Col4a3* mutant/Alport syndrome mouse model. Our study revealed unprecedented details of glomerular abnormalities in *Col4a3* mutants including distorted podocyte cell bodies and disorganized primary processes. Strikingly, we observed abundant filamentous microprojections arising from podocyte cell bodies and processes, and presence of unique bridging processes that connect the primary processes and foot processes in Alport mice. Furthermore, we detected an altered glomerular endothelium with disrupted sub-endothelial integrity. More importantly, we were able to clearly visualize the complex, three-dimensional podocyte and endothelial interface by HIM. Our study demonstrates that HIM provides nanometer resolution to uncover and rediscover critical ultrastructural characteristics of the glomerulopathy in *Col4a3* mutant mice.

## Introduction

Ultrafiltration of plasma through a complex glomerular filtration barrier, consisting of podocytes, endothelial cells and glomerular basement membrane (GBM), represents the most important function of the kidney^1,2^. Podocytes are highly specialized cells that serve as major architectural components of the filtration barrier. They are composed of a cell body, several primary and secondary processes, and numerous foot processes, that protrude from each primary or secondary process^3^. The slit diaphragm (SD) is a unique, specialized intercellular adhesion structure formed by interdigitating foot processes from adjacent podocytes^4^. The SD is a highly organized and dynamic structure, that contains a number of proteins including the well-known key SD proteins CD2-associated protein (CD2AP), nephrin, and podocin, as well as several SD-associated proteins, such as atypical protein kinase C (aPKC), Ras GTPase-activating-like protein IQGAP1, membrane-associated guanylate kinase inverted 2 (MAGI-2), and zona occuldens-1 (ZO-1)^5–10^. Disruption of most SD components leads to glomerulopathy and frequently proteinuria^11^. In addition, another critical component of the glomerular filtration barrier is the GBM. The GBM forms a complex extracellular matrix (ECM) network not only providing mechanical support to podocytes and endothelial cells but also contributing directly to the permselectivity of the glomerular filtration barrier^12^. In addition, the GBM is critical for epithelial cell organization, survival and function^13^.

The GBM is initially derived from the fusion of the basement membranes of podocytes and endothelial cells. It consists primarily of type IV collagen α3α4α5 and laminin α5β2γ1, alongside many other proteins^14,15^. In the mature kidney, *COL4A3, COL4A4*, and *COL4A5* are expressed by the podocyte and are required for the assembly of the major type IV collagen network of the GBM. Mutations in *COL4A5* cause the X-linked form of Alport syndrome, a hereditary glomerulonephritis that is associated with hearing and ocular defects^16^. Mutations in *COL4A3* and *COL4A4* cause the autosomal recessive forms of the disease, as well as thin basement membrane nephropathy and an FSGS-like pathology^17,18^. The pathological characteristics of Alport syndrome in the kidney include changes from thinning to mixed thinning and thickening and splitting of the GBM, often described as a ‘basket weave’ appearance. More often at the later stage there is a reduction in podocyte number and effacement of podocyte foot processes^19,20^. Here we designed a study using a novel microscopy technology to investigate the complex ultrastructure of the Alport glomerulopathy associated with COL4A3 deficiency^13^.

It is well known that the podocytes, GBM and associated endothelium constitute a glomerular filtration apparatus of very complex three-dimensional architecture^3^. Studying this structure by conventional Scanning Electron Microscopy (SEM) has provided critical insights into glomerular biology, pathophysiology, and the underlying mechanisms of kidney diseases^21–23^. For example, Makino *et al*. visualized gaps in the GBM and red blood cells passing through these gaps in hematuric animals with a combination of transmission electron microscopy (TEM) and SEM, thus uncovering the cause of hematuria associated with kidney disease^24^. Foot process effacement detected by TEM and SEM has become a hallmark of proteinuric glomerulopathy/podocytopathy^25^. Constantly improving conventional SEM technology has allowed the detection of numerous key ultrastructural features of the healthy and diseased glomerulus, thus significantly advancing our understanding of glomerular biology, physiology, and pathophysiology. More recently, through applying block-face scanning electron microscopy and image reconstruction, the sub-podocyte expansion/invasion into the GBM was revealed in Alport syndrome animals^26^. Another study using the same technology uncovered the presence of a “ridge like prominence” formed on the basal surface of the primary process that attaches to the GBM^27^. However, despite these exciting discoveries, the widely used conventional SEM technology has been greatly limited by the imaging resolution at high magnification due to charging interference caused by the insulating properties of tissues and the loss of subtle surface features due to heavy metal coating^28^.

Remarkable progress has been made during this decade in research of glomerular diseases, particularly in cell and molecular biology. This highlights the need for powerful microscopic technologies required to enable the detection of sophisticated cellular and/or molecular events, and possibly to characterize molecular anatomic details of cells and subcellular structures at nanometer resolution scale. However, conventional microscopic technologies seem to have reached their technical capacity and to no longer be able to fill in knowledge gaps. Excitingly, the recently developed high resolution Helium Ion scanning Microscopy (HIM) offers unique advantages over conventional SEM through reduced sample charging, minimizing sample damage, and providing better surface contrast without metal coating^29–31^. Importantly, it enables an increased depth of field and potentially 5 angstrom imaging resolution. Scanning HIM has recently been used by our group to visualize the ultrastructure of the kidney from normal rodents with nanometer resolution^28^. In the current study, we apply this scanning HIM technique to examine glomerular abnormalities in the collagen type IV α3 chain (*Col4a3)* deficient mice that model Alport syndrome. We applied HIM to examine the three-dimensional ultrastructural details of the glomeruli, focusing on the morphology of the podocyte cell body, primary processes and foot processes, filtration slits, endothelial surface, as well as the interface between podocyte and endothelium in heterozygous and homozygous animals.

## Results and Discussion

### Three-dimensional view of podocytes in *Col4a3* mutant mice

**Figure 1:**
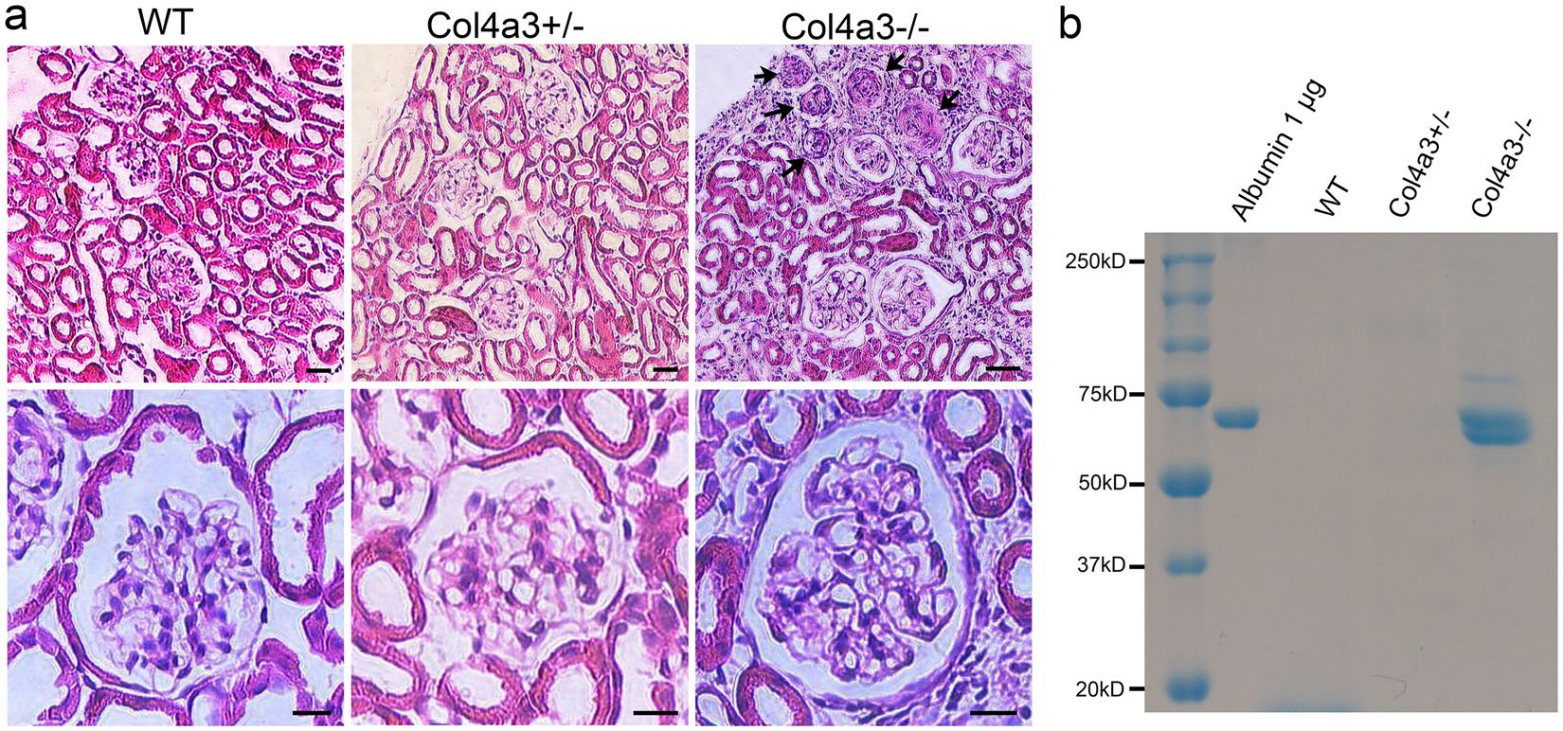
Analysis of proteinuria and kidney histology. (**a**) Representative images of H&E staining of WT, *Col4a3*+/−, and *Col4a3*−/− kidney sections. Glomeruli in wild-type (WT) and *Col4a3*+/− kidneys have a normal appearance. The right upper panel shows several sclerosed glomeruli (arrows) along the surface of a cortical lesion and interstitial injury in a *Col4a3*−/− kidney. Scale bars, 50 μm in upper panels; 10 μm in lower panels. (**b**) Coomassie blue staining reveals a strong albumin band in spot urines collected from *Col4a3*−/− mice but not from the WT and *Col4a3*+/− mice.

4–5 month old *Col4a3*−/− (Alport) mice lacking the collagen α3α4α5(IV) network and wild-type (WT) and heterozygous (*Col4a3*+/−) control mice were used for scanning HIM. Spot urine samples from all animals were collected for analysis of proteinuria by SDS-PAGE. Coomassie blue staining detected the presence of significant amounts of albumin in urine samples from homozygous *Col4a3*−/− mice, but not from *Col4a3*+/− mice (Fig. 1b, Figure S1). H&E staining of kidney sections revealed that there were no obvious abnormalities of the glomerular structure in the *Col4a3*+/− animals compared to WT controls (Fig. 1a). In the *Col4a3*−/− mice, about 10% of glomeruli appeared sclerosed, and the remaining glomeruli appeared grossly intact. These animals also had increased ECM deposition and cellular components in the interstitium. These results are consistent with a previous report on these mice^32^.

**Figure 2:**
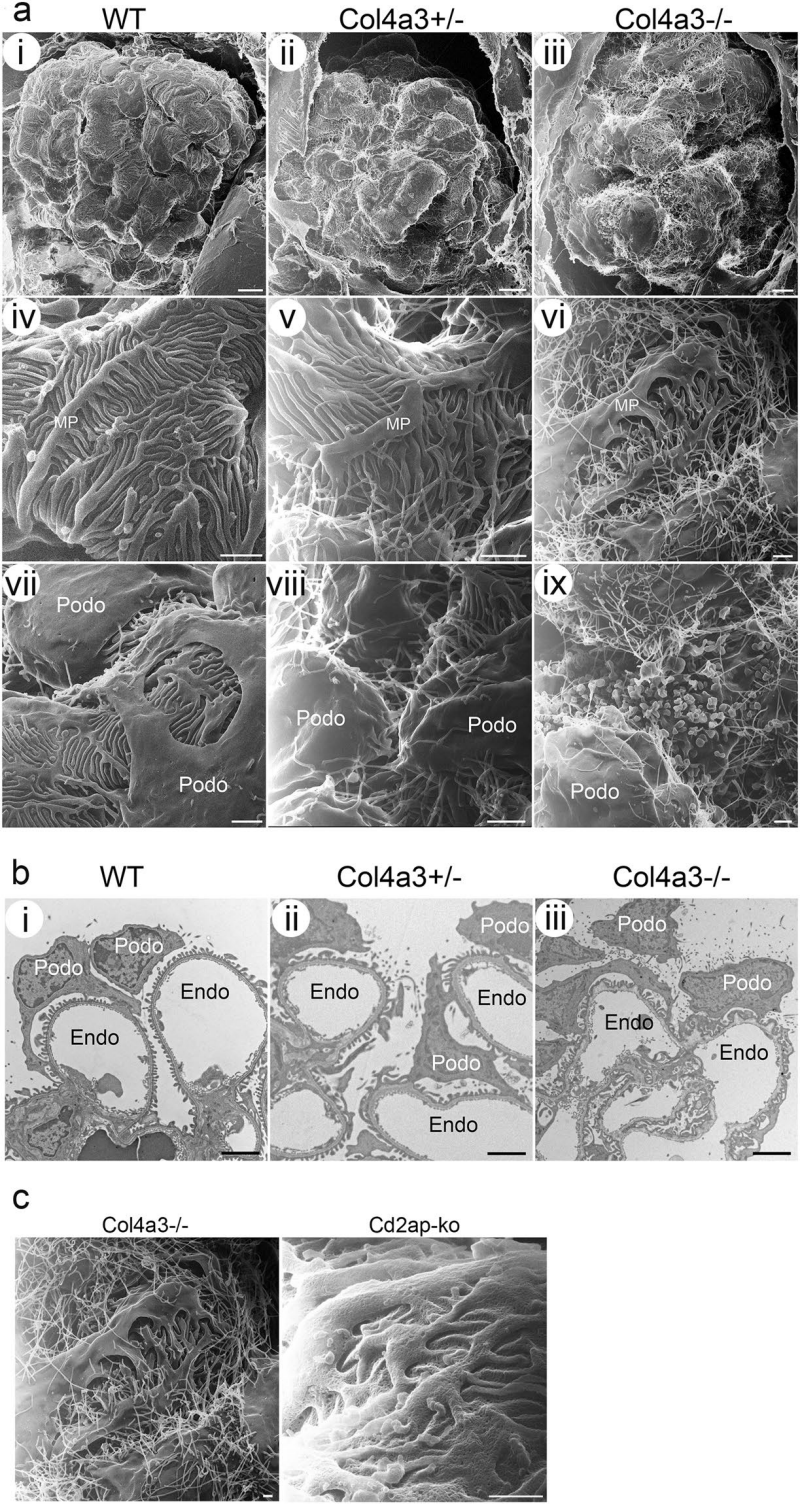
Glomerular and podocyte morphology in wild-type and *Col4a3* mutant mice. (**a**) HIM images of glomeruli in wild-type (WT) (**i**,**iv**,**vii**), *Col4a3*+/− (**ii**,**v**,**viii**), and *Col4a3*−/− mice (**iii**,**vi**,**ix**). Low magnification image of WT kidney (**i**) shows the glomeruli formed of capillary loops covered with podocytes while images of *Col4a3*+/− (**ii**) and *Col4a3*−/− kidneys (**iii**) show a less regular and less smooth surface of podocytes. High magnification images of WT kidneys (**iv**,**vii**) show multiple thick primary or major foot processes projecting from the podocyte cell body and covering the capillary loops. The primary processes frequently cross over each other, and numerous uniformly shaped underlying foot processes arise from the primary processes forming an interdigitated network. There are rounded and filamentous microprojections on the free surface of the podocyte cell body and primary processes (**vii**). The primary processes are flattened, broader and less organized, and the foot processes more randomly branched in *Col4a3*−/− kidneys (**vi**) compared to WT. An increased number of longer microprojections, arising from primary processes and foot processes, was detectable in *Col4a3*+/− (**v**,**viii**) and *Col4a3*−/− kidneys (**vi**,**ix**). Numerous “blebs” projecting out of the epithelial surface between podocyte cell bodies could be observed (**ix**). Scale bars, 5 μm in upper panels; 1 μm in middle panels and lower panels. (**b**) Representative TEM images of WT (**i**), *Col4a3*+/− (**ii**), and *Col4a3*−/− glomeruli (**iii**). WT glomeruli (**i**) show normal GBM thickness and regularly lined-up foot processes. *Col4a3*−/− glomeruli (**iii**) show irregularly thickened and lamellated GBM. Some podocytes have widened foot processes and SDs disappear between interdigitating foot processes. Increased microvilli formations are detectable. Scale bars, 2 μm. MP, major process; Podo, podocyte; Endo, endocapillary space. (**c**) Microprojections in *Col4a3* and *Cd2ap* knockout (ko) mice. HIM images of glomeruli in *Col4a3*−/− (left panel, copied from Fig. 2a-vi, for comparison) and *Cd2ap-ko* mice (right panel). While an increased number of longer microprojections arise from primary processes and foot processes in *Col4a3*−/− kidneys, they are less obvious in *Cd2ap-*ko kidneys. Scale bars, 500 nm.

By low magnification HIM examination, the WT glomeruli appeared well organized with the podocyte cell body sitting above the capillary loops. Multiple thick primary/major foot processes projected from the podocyte cell body and covered the underlying glomerular capillaries. The primary processes were frequently found crossing over each other and under the podocyte cell body forming a complex network. Arising from the major processes were numerous smaller, uniformly shaped foot processes oriented in a fern like pattern (Fig. 2a-i,iv,vii). Foot processes from adjacent podocytes interdigitated to form filtration pores that completely cover the underlying capillaries. We also observed the sporadic appearance of rounded and filamentous microprojections on the free surface of the podocyte cell body and major processes in WT kidneys. HIM revealed that the surface of the *Col4a3*−/− and *Col4a3*+/− podocytes was less regular and less smooth compared to WT podocytes (Fig. 2a-ii,iii). The major processes were flattened, broader and less organized (Fig. 2a-vi) and the foot processes branched more randomly than in WT kidneys.

We compared scanning HIM with TEM. By TEM, WT glomeruli showed an intact GBM with a discrete layered structure, and well organized foot processes lined up around the capillary loop. All foot processes were regularly spaced and attached to the GBM. A fine diaphragm structure between interdigitating foot processes was clearly visible (Fig. 2b-i). In the *Col4a3*−/− glomeruli, in some parts of the glomerulus the GBM was irregularly thickened, lamellated and protruding out towards the basal surface of the podocytes (Fig. 2b-iii). This GBM abnormality was interspersed with areas of normal appearing GBM. Similarly, some podocytes appeared ultrastructurally normal while others had widened foot processes, and SDs disappeared between interdigitating foot processes (Fig. 2b-iii).

One very striking morphological feature of the *Col4a3*−/− glomeruli imaged by HIM was the presence of numerous long filamentous microprojections. These projections were so abundant that they formed a “hairy web” covering the entire glomerulus (Fig. 2a-iii,vi). Also of note was the appearance of numerous “blebs” projecting out of the epithelial surface in between podocyte cell bodies (Fig. 2a-ix). Filamentous projections were also observed in *Col4a3*+/− mice despite those animals lacking proteinuria and having grossly normal kidneys by H&E staining (Fig. 2a-v,viii). Increased microprojections were also observed by TEM in *Col4a3*+/− and *Col4a3*−/− glomeruli (Fig. 2b-ii,iii); HIM revealed the high-resolution ultrastructural details of the global disorganization of podocyte processes and abundant distribution of microprojections in *Col4a3* mutant kidneys. These increased filamentous microprojections were only observed in the Col4a3 mutant kidney, and not in another glomerulopathy model of *Cd2ap*-knockout mice (Fig. 2c), which is a genetic model known to cause podocyte injury^33^, suggesting that the “hairy web” structure with numerous microprojections is likely to be unique to *Col4a3*+/− mice. The increased abundance of the filamentous and bleb-like (round shaped) projections in *Col4a3* mutant glomeruli might indicate an increased cellular response of podocytes to signals and/or injury induced by the GBM defect. Indeed, the presence of microprojections was previously observed in the context of cell differentiation^34^, aging, and disease states such as nephrotic syndrome^35,36^ by TEM and conventional SEM. However, their abundance and the clarity of their structure have never been appreciated to the degree shown here by HIM. The biological and pathological implications of these microprojections remain to be elucidated.

### Foot processes and filtration slits in *Col4a3* mutant glomeruli

**Figure 3:**
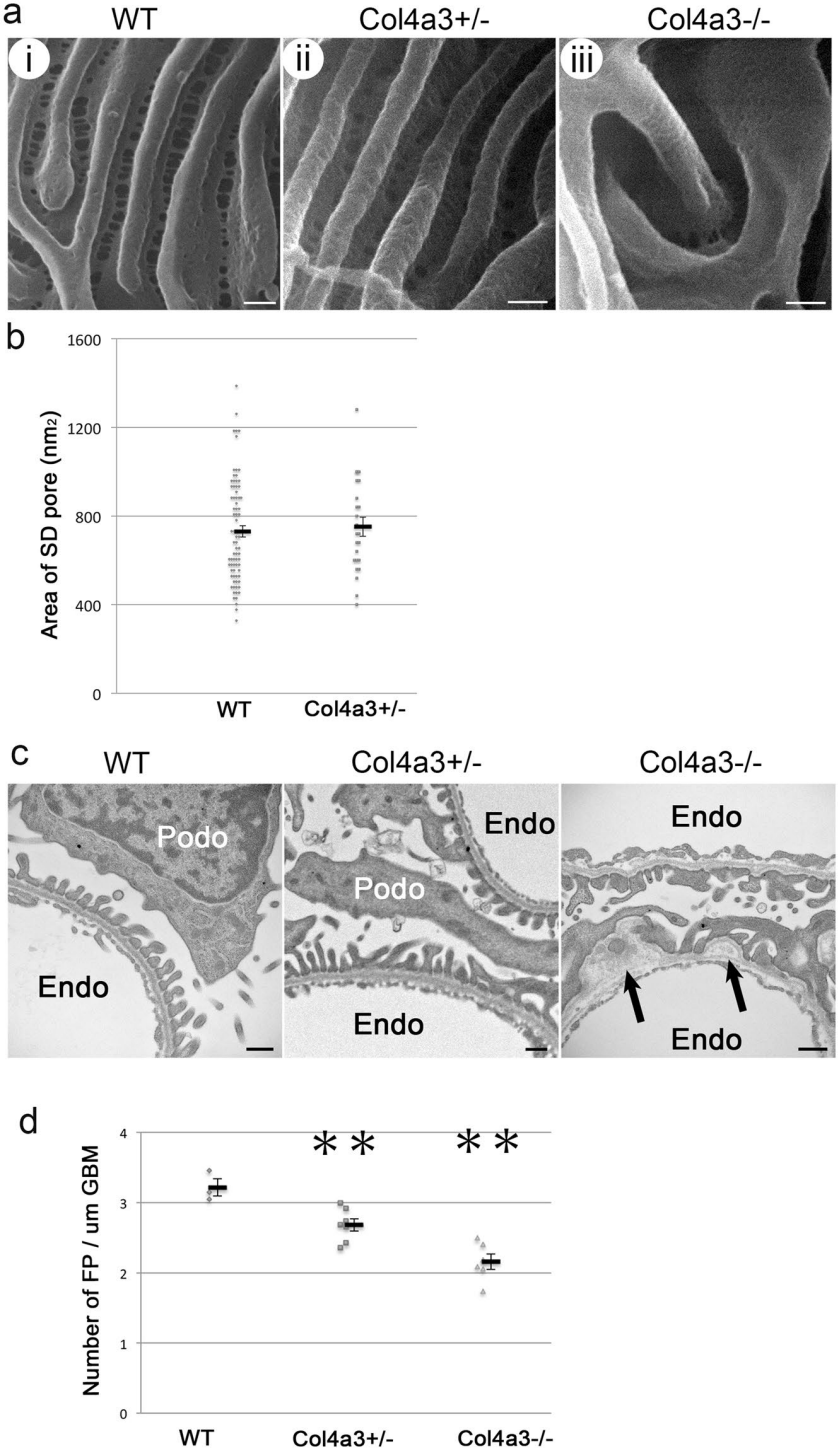
Foot processes and filtration slits in wild-type and *Col4a3* mutant mice. (**a**) HIM images of glomerular filtration slits in wild-type (WT) (**i**), *Col4a3*+/− (**ii**), and *Col4a3*−/− mice (**iii**). The WT kidney image (**i**) shows filtration regions between foot processes. The image of the foot processes in *Col4a3*+/− kidney (**ii**) shows a similar pattern to WT. In the *Col4a3*−/− kidney (**iii**), the slit pores are difficult to visualize due to the depth of the foot processes. Scale bar, 100 nm. (**b**) The size of the SD pores measured from HIM images in WT (n = 78) and *Col4a3*+/− (n = 24) mice shows no significant difference as assessed by Student’s *t*-test. Values are presented as means ± standard error of the mean (SEM) here and in the following plots. (**c**) Representative TEM images of WT, *Col4a3*+/−, and *Col4a3*−/− glomeruli. WT and *Col4a3*+/− glomeruli show normal GBM and associated foot processes. In *Col4a3*−/− glomeruli, the GBM was irregularly thickened and lamellated (arrow). Scale bar, 500 nm. Podo, podocyte; Endo, endocapillary space. (**d**) The number of foot process (FP) per unit GBM length (μm) is significantly lower in *Col4a3*+/− (n = 7) and *Col4a3*−/− (n = 6) compared to WT (n = 3) (**p < 0.01 by Student’s *t*-test). Each analysis includes approximately 10 μm GBM length.

HIM enables direct visualization of foot processes and filtration slits with unsurpassed resolution. The foot processes of WT and *Col4a3*+/− animals were mostly of uniform size and well organized (Fig. 3a-i,ii). They appeared wider in *Col4a3*−/− mice (Fig. 3a-iii), which is consistent with our observations by TEM. We detected a slight but significant decrease in the number of foot processes per length of GBM in *Col4a3*−/− mice, which is consistent with the occurrence of foot process effacement in these animals as shown by TEM (Fig. 3,c and d).

Despite the difficulties in visualizing filtration slits in *Col4a3*−/− glomeruli due to the deeply located foot processes and the presence of many overlaying “bridging” processes above the foot processes, we found that the visible filtration slits were grossly intact in both *Col4a3*+/− and *Col4a3*−/− mice (Fig. 3a-ii,iii). There was no significant difference in SD pore area measured in HIM images between WT and *Col4a3*+/− mice (Fig. 3b). It was difficult to measure the SD pore area in *Col4a3*−/− mice due to the deeply located foot processes (Fig. 3a-iii). Overall, based on data from both HIM and TEM, the filtration slit does not seem to be significantly altered in *Col4a3*−/− mice, suggesting that the GBM composition is unlikely to directly modify the filtration SD structure. This is consistent with a previous report that laminin β2 deficiency results in proteinuria without a significant alteration of SD structure^12^. Our data are also consistent with a recent study in Alport syndrome animals using serial block-face scanning electron microscopy^26^.

### Bridging processes in *Col4a3* mutant glomeruli

**Figure 4:**
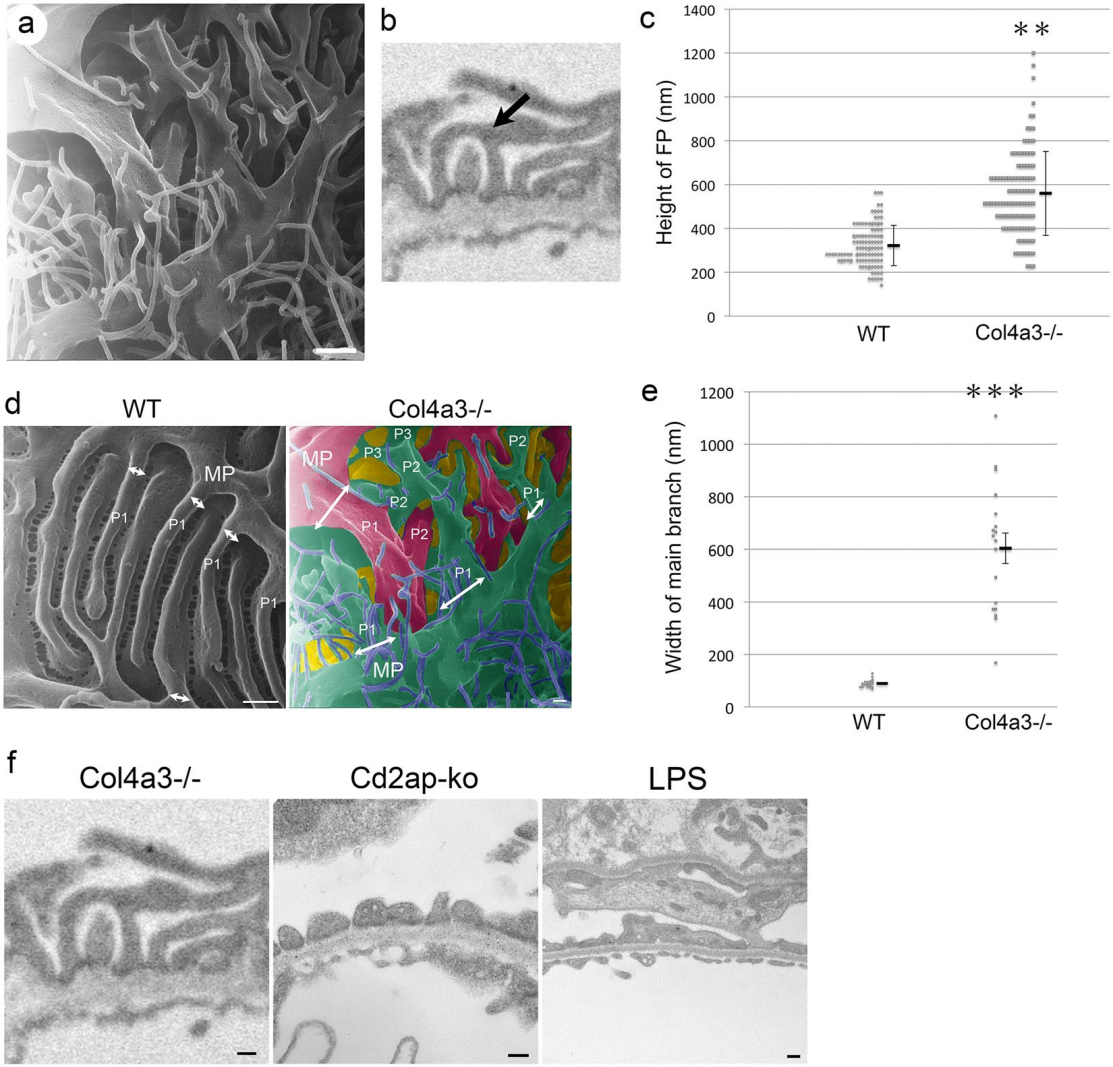
Increased bridging processes in *Col4a3*−/− glomeruli. (**a**) Representative image of *Col4a3*−/− glomeruli shows primary processes giving rise to many “intermediate” processes with more branching formations. These intermediate processes intercross with each other and form “bridge”-like structures between primary and foot processes. Scale bar, 500 nm. (**b**) Representative image of foot processes of *Col4a3*−/− glomeruli in TEM. Arrow indicates a bridging foot process. (**c**) The foot process (FP) height is significantly increased in *Col4a3*−/− mice (n = 108) compared to wild-type (WT) mice (n = 114) (**p < 0.01 by Student’s *t*-test). (**d**) HIM images of podocyte processes in wild-type (WT) (left panel) and *Col4a3*−/− (right panel) mice. White arrows represent the width of main branch from major processes. Pseudo-colored picture was obtained by processing Fig. 4a in Adobe Photoshop (right panel). The pseudo green color represents podocyte major process and foot processes from a podocyte, and the pseudo red color represents podocyte major process and foot processes from another podocyte. Pseudo purple color represents microprojections originating from podocytes. MP, major processes; P1, main branch from MP; P2, second branch; P3, third branch. Scale bars, 200 nm. (**e**) The width of main branch from podocyte major processes is significantly increased in *Col4a3*−/− (n = 18) compared to WT (n = 23) glomeruli (***p < 0.001 by Student’s *t*-test). (**f**) Representative TEM images of processes in *Col4a3*−/− (left panel, copied from Fig. 4b, for comparison), *Cd2ap-knockout (ko)* and lipopolysaccharide (LPS) treated mice (right panel). While bridging processes are observed in *Col4a3*−/− glomeruli, diffuse foot process effacement and flattened foot processes are detected without bridging processes in *Cd2ap*-ko and LPS-treated glomeruli. Scale bars, 200 nm.

Another interesting observation was the presence of numerous tall and arched processes projecting from the primary processes in *Col4a3*−/− glomeruli (Fig. 4a). In the *Col4a3*−/− glomeruli, the primary processes gave rise to many “intermediate” processes rather than foot processes. These intermediate processes intercrossed with each other and formed “bridges” between primary and foot processes (Fig. 4b). These “bridging” processes did not appear to attach directly to the GBM but rather to overlying foot processes. The bridging processes seemingly gave rise to foot processes that attached to the GBM (Fig. 4a). We have observed previously that the height of podocyte foot processes measured by TEM in *Col4a3*−/− kidney was significantly larger than that in WT kidney (Fig. 4c). This is probably due to the formation of the arched bridging processes. Indeed, a careful examination revealed that bridge-like processes were present in over 7% of foot processes in *Col4a3*−/− mice (39 foot processes with bridge shape out of 550 foot processes) compared to only 0.8% seen in WT animals (2 foot processes with bridge shape out of 250 foot processes) by TEM. We also noticed an increased presence of the “bridging processes” in *Col4a3*+/− glomeruli (12 foot processes with bridge shape out of 605 foot processes). The pseudo-colored picture was created to allow for the easier visualization of complex structural details in *Col4a3*−/− glomeruli (Fig. 4d, right panel), and it revealed more branching of podocyte processes from major processes compared to WT control (Fig. 4d, left panel). Furthermore, the width of branches derived from the major processes is larger in *Col4a3*−/− podocytes compared to WT podocytes (Fig. 4d). Quantitative analysis revealed that the width of the first branches coming out of the major processes was dramatically increased (Fig. 4e), and moreover there are multiple small branches coming out of the major processes in a step wise manner in *Col4a3*−/− glomeruli compared to WT (Fig. 4d).

We then examined other glomerulopathy models, *Cd2ap*-knockout mice and lipopolysaccharide (LPS) treated mice, an acquired model of podocyte injury^37^, for the presence of the bridging process seen in the *Col4a3*−/− mouse (Fig. 4b). We observed diffuse foot process effacement and flattened foot processes in *Cd2ap*-knockout and LPS treated glomeruli. None of them had bridging processes (Fig. 4f). We have not found a description of similar “bridging processes” in the literature either. There was a recent report of “ridge-like prominences” observed by block-face scanning electron microscopy^27^. These “ridge-like prominences” project from the basal side of the podocyte cell body and major processes, and are closely attached to the GBM along the foot processes. Functionally, they were proposed to provide additional adhesion to the GBM^27^. The bridging processes that we observed are not attached to the GBM and therefore are likely not the same entity as the “ridge-like prominences”. The ultrastructural abnormalities we described above are seen in nearly 90% of glomeruli in *Col4a3*−/− mice at the age of 4–5 months. In the approximately 10% glomeruli that are severely sclerosed, this described pathology was no longer detectable.

The functional significance of the “bridging processes” we observed is unknown. We hypothesize that these processes may be a compensatory mechanism to increase the branching of foot processes, thereby enhancing the overall adhesion of the foot processes to the GBM, or a pathological outcome resulting from a GBM defect due to the lack of COL4A3. This bridging process was not observed in podocytes from other glomerulopathy models, implicating that it is a unique feature of the *Col4a3* mutant glomeruli.

It is well known that defects in COL4A3, COL4A4 or COL4A5 disrupt the assembly of type IV collagen α3α4α5 heterotrimer, inducing instead the formation of the α1α1α2 heterotrimer complex in the Alport syndrome glomeruli^38^. It was reported that collagen α1 and α2 chains have fewer cysteines, and hence cross-link at a lesser extent within and between the heterotrimers^39,40^. Recent analysis revealed that type IV collagen is normally located in the center of the GBM and, as such, is too remote from the podocyte to mediate the integrin-ECM interaction. However, the α1α1α2 collagen is mislocalized in Alport syndrome and becomes adjacent to podocytes^41^, and, therefore, alters the cell-ECM interaction via integrin-mediated signaling. Replacement of normal type IV collagen with α1α1α2 heterotrimers results in ectopic overexpression of laminins α1 and α5 and disrupts the GBM architecture^38^. A growing body of evidence reveals a complex interplay between podocyte, endothelium, GBM and integrins in glomerular physiology and pathophysiology^42–45^. The podocyte senses the altered composition of collagen type IV and/or laminins through the α2β1 integrin receptor^38^. Alteration of integrin signaling is well known to affect its downstream targets such as the integrin-linked kinase (ILK) and small Rho GTPases including RhoA and Rac1, and results in reorganization of microtubules and actin cytoskeleton network^46–48^. For example, a recent study by Dr. Reiser’s group has revealed an essential role of the soluble urokinase-type plasminogen activator receptor (suPAR) in the pathogenesis of focal segmental glomerulosclerosis (FSGS)^49^. Upon binding to αvβ3 integrin, suPAR activates the integrin downstream effector, small GTPase Rac1, thus resulting in foot process effacement and proteinuria via rearrangement of microtubules and actin cytoskeleton network^49^. The podocyte foot processes are enriched with actin and myosin, and the podocyte primary processes contain an important population of microtubules and microfilaments, both subjected to remodeling in response to the ECM-integrin signaling. In addition to small GTPases, the large GTPase dynamin has recently been shown to regulate the actin cytoskeleton in podocytes^50^. The small molecule Bis-T-23, which promotes actin-dependent dynamin oligomerization, ameliorates proteinuria in multiple kidney disease animal models^51^. Whether the newly uncovered bridging processes are subjected to dynamic regulation of microtubule and microfilament rearrangement by ECM-integrin signaling, and small or large GTPases, remains to be elucidated.

### Podocyte ultrastructure at late stages of disease in *Col4a3* mutant mice

**Figure 5:**
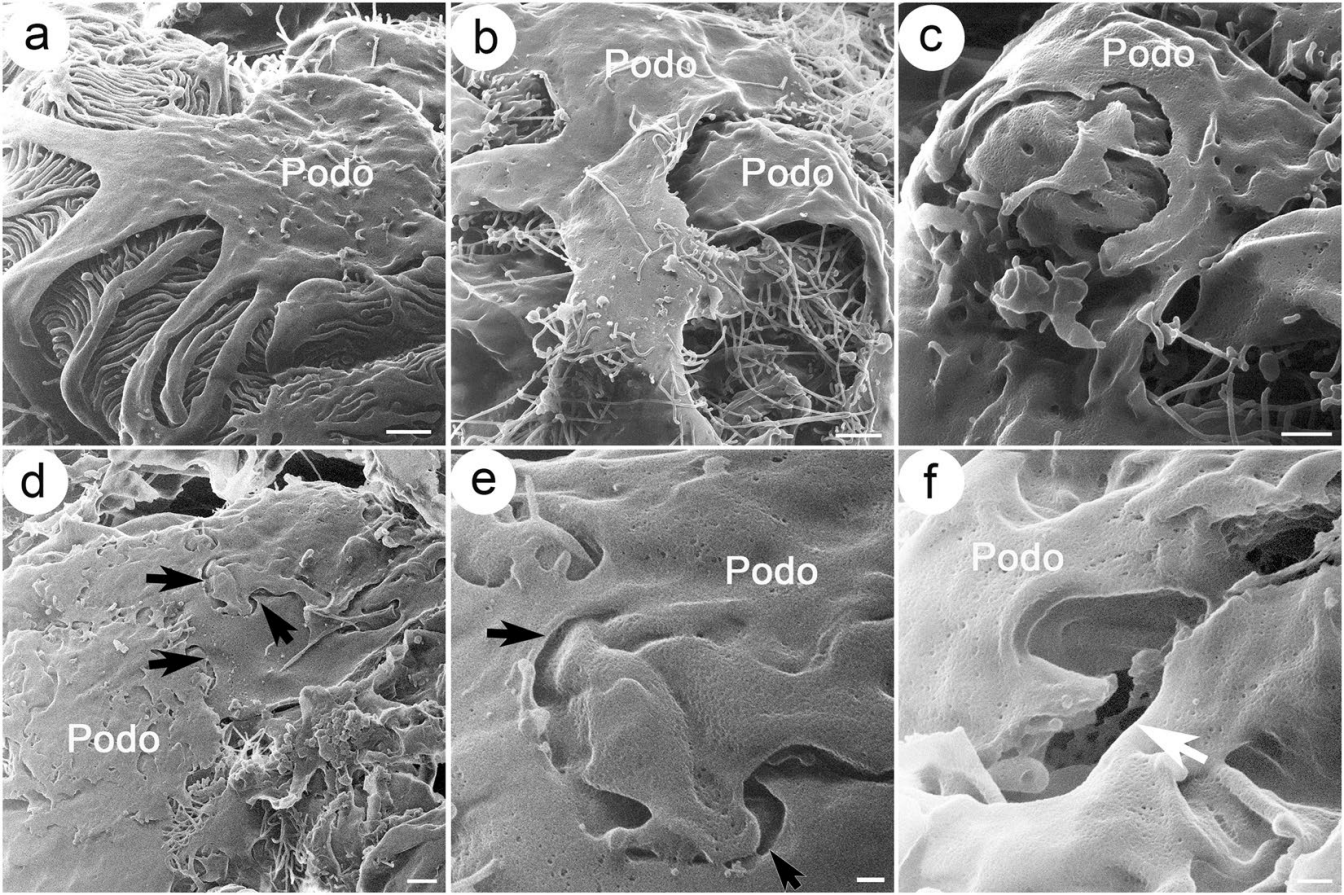
Podocyte ultrastructure at late stage in *Col4a3* mutant mice compared with wild-type mice. Shown are HIM images of podocytes at late stage in wild-type (WT) (**a**) and *Col4a3*−/− mice (**b**–**f**). (**a**) WT podocytes show intact podocyte structure with major processes and foot processes forming interdigitated structures. Late stage *Col4a3*−/− podocytes show broadly effaced podocyte processes that form large flattened sheets covering capillary walls (**b**,**c**). In some regions, podocytes appear connected by junctional structures (arrows) (**d**,**e**). In other regions, the presence of a few breaks/holes and gaps between podocytes is detected (white arrow) (**f**). A mesh structure appearing as degraded endothelial fenestrae is visible in the gap. Scale bars, (**a**,**b**,**d**) 1 μm; (**c**) 500 nm; (**e**,**f**) 200 nm. Podo, podocyte.

In the late stage glomeruli in *Col4a3*−/− mice, HIM revealed that the distinction between primary and foot process morphology as seen in the WT animals (Fig. 5a) was lost and was replaced by broadly effaced podocyte processes that formed large and flattened sheets covering capillaries below. Some of the “sheets” crossed over each other (Fig. 5b–e). In some regions they were connected by junctional structures (arrows in Fig. 5,d and e). We did not observe complete detachment or denudation of podocytes from the GBM, which could account for the presence of proteinuria in non-terminally sclerosed kidney. However, from time to time, we did observe the presence of a few breaks/holes and gaps between podocytes, and fragmentation of podocyte sheets (arrow in Fig. 5f). It is unclear whether these holes and gaps and sheet fragmentation are intrinsically formed due to mechanical defects, or generated during the sample preparation process. However, they have not been observed in WT kidneys subjected to the same procedure.

### Alteration of endothelium and podocyte and endothelial interface in *Col4a3* mutant glomeruli

**Figure 6:**
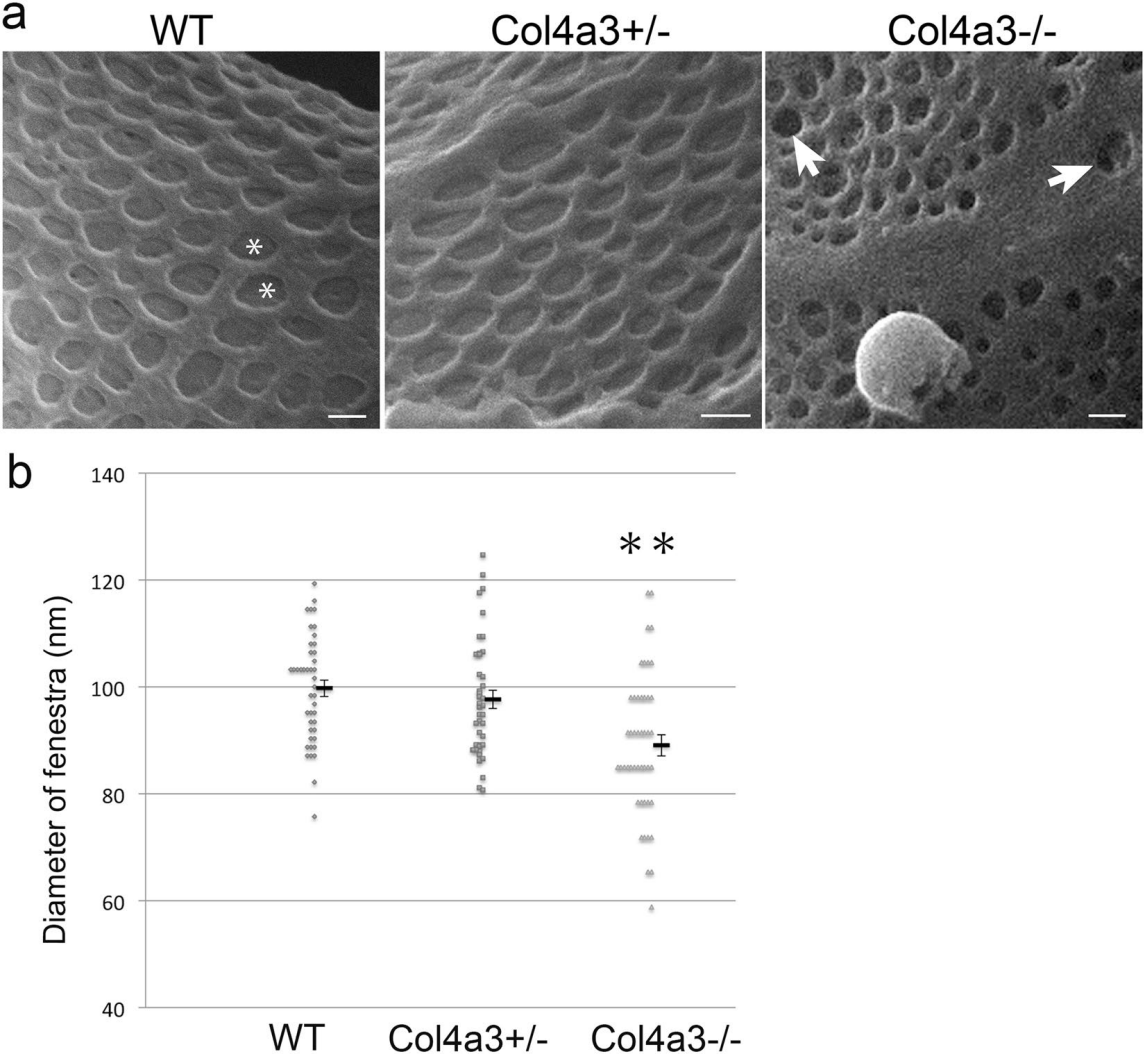
Endothelium in wild-type and *Col4a3* mutant mice. (**a**) HIM images of glomerular filtration slits in wild-type (WT), *Col4a3*+/−, and *Col4a3*−/− mice. The image of WT endothelium shows a smooth endothelial surface and endothelial fenestrae of regular size. Underlying the endothelial fenestrae, a diaphragm like structure is detectable (*). The image of *Col4a3*+/− endothelium shows a similar pattern to WT. The *Col4a3*−/− endothelium shows irregular sizes of endothelial fenestrae and disappearance of the diaphragm-like structure underneath the fenestrae (white arrows). Scale bars, 100 nm. (**b**) The diameter of endothelial fenestrae is significantly decreased in *Col4a3*−/− (n = 46) mice but not in *Col4a3*+/− (n = 41) mice compared to WT (n = 43) (**p < 0.01 by Student’s *t*-test).

**Figure 7:**
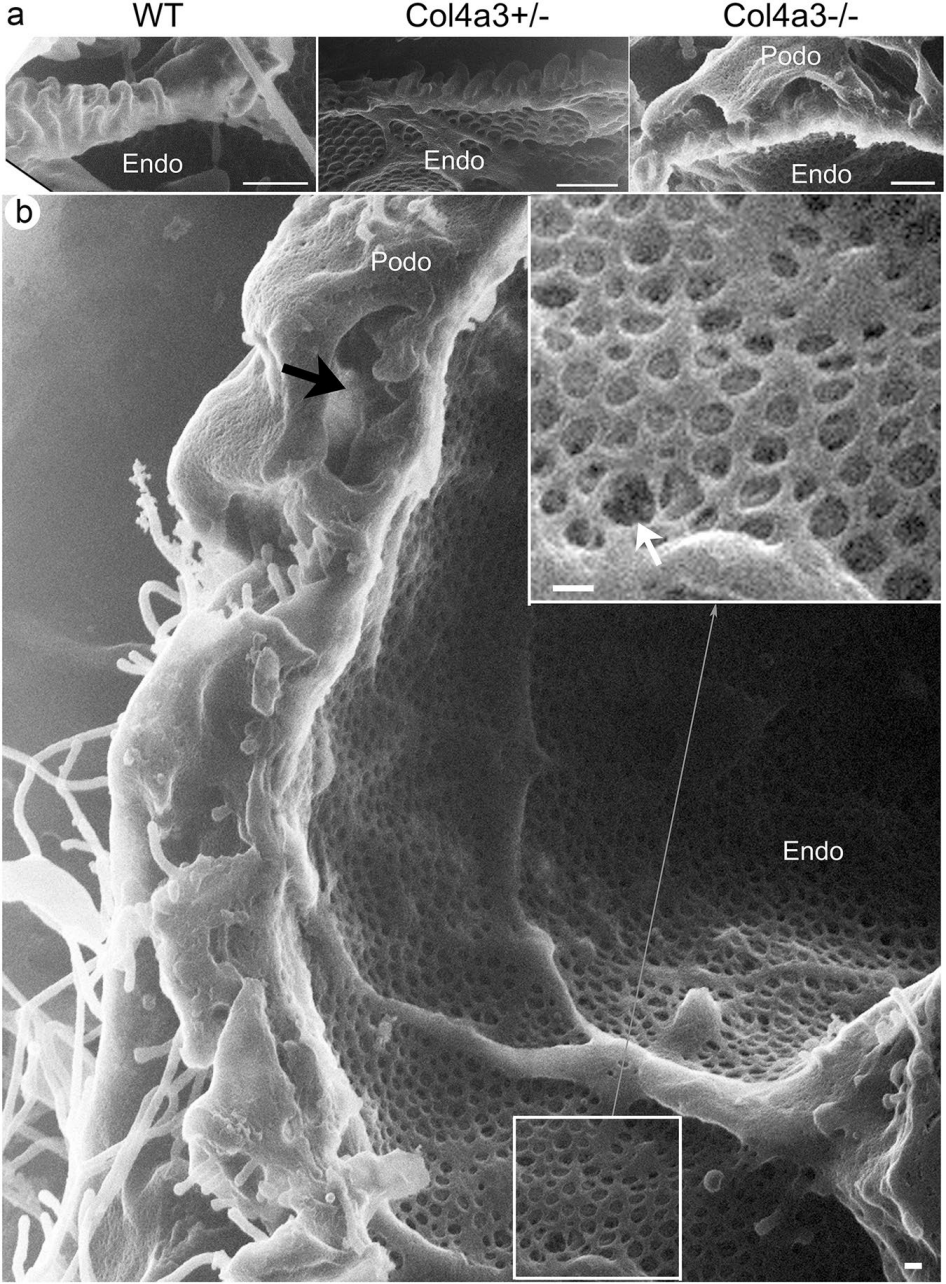
Interface between podocyte and endothelium in wild-type and *Col4a3* mutant mice. (**a**) HIM images of the interface between podocytes and endothelium in WT, *Col4a3*+/−, and *Col4a3*−/− mice. The WT image shows well organized foot processes lining the capillary loops. A similar pattern is seen in *Col4a3*+/− mice. In *Col4a3*−/− mice, foot processes appear effaced and form flat sheets covering the GBM. “Bridging” process structures are detectable underneath the podocyte cell body. Scale bars, 500 nm. (**b**) Transverse image of the interface between podocytes and endothelium in *Col4a3*−/− kidney shows largely effaced foot processes and bridging process structures. The bridging processes and flattened podocyte cell body arched over effaced foot processes are indicated by a black arrow. The disappearance or fragmentation of the diaphragm-like structure underneath the fenestrae is indicated by a white arrow. Scale bar, 100 nm.

Whether the GBM defect affects endothelial structure cannot be determined by conventional SEM, although evidence obtained by TEM suggests it in rodents and patients with glomerulopathy^52–54^. Remarkably, HIM allows the endothelial surface to be directly and clearly visualized (Figs 6 and 7). In WT mice, the endothelial surface was smooth, and the endothelial fenestrae were regular and well organized (Fig. 6a). Underlying the endothelial fenestrae was likely the endothelial aspect of the GBM. It formed a smooth membranous sheet underneath the fenestrae (asterisk in Fig. 6a). Equally well visualized was the endothelial surface in *Col4a3*+/− mice that appeared without gross defects (Fig. 6a). However, in *Col4a3*−/− mice, we detected a seemingly thickened endothelium with irregularly shaped and sized endothelial fenestrae (Fig. 6a). Strikingly, many of the endothelial fenestrae lost the underlying supporting structure. In some endothelial fenestrae, some deep “holes” underneath were clearly seen suggesting a disruption of the underlying GBM (white arrows in Fig. 6a). A significant reduction of the size of endothelial fenestrae in *Col4a3*−/− glomeruli was confirmed by quantitative analysis of HIM images (Fig. 6b). Taken together, our data suggest the presence of endothelial defects in the *Col4a3*−/− glomeruli. Since we observed this endothelial alteration more commonly in the glomeruli at the more advanced disease state, this is likely a feature of the late stage.

A transverse view of the interface between podocytes and endothelium could also be obtained by HIM (Fig. 7a). In the WT glomerulus, well-organized foot processes separated by the filtration SD were lined up along the capillary wall (Fig. 7a). Similarly well-organized foot processes were observed in *Col4a3*+/− mice (Fig. 7a). Conversely, in *Col4a3*−/− glomeruli, foot processes largely disappeared, becoming effaced and forming flat sheets covering the GBM (Fig. 7a). The previously observed “bridging” process structures were seen underneath the podocyte cell body and arching over the GBM. Again, they did not seem attached to the GBM (Fig. 7a). Interestingly, according to the transverse view, the bridging processes and flattened podocyte cell body arched over some effaced foot processes (Fig. 7a). Thus, they created a false “cyst-like structure” that is reminiscent of the “intra-podocyte cysts/vacuoles” that were frequently seen by cross-sectioned TEM^55–57^. Therefore, the previously reported intra-podocyte cysts seen by two-dimensional TEM in many glomerulopathies may well be formed by the very complex crossing over of 3D structures as revealed by HIM. However, what was clearly seen was the presence of many “holes” and loss of underlying membranous structure of the fenestrae (Fig. 7b), suggesting disrupted GBM underneath the endothelium.

Recent three-dimensional block-face SEM has revealed the presence of podocyte invasion into the GBM in Alport nephropathy models and suggested the interaction between podocyte structures and the GBM^26^. We analyzed 4–5 month old *Col4a3*−/− mice. Through directly visualizing their surface structure, we were unable to conclude whether podocytes actively invade the GBM in Alport glomerulopathy or not. We did not detect signs of active migration of podocytes in *Col4a3*−/− mice either. More comprehensive HIM incorporating microstructuring technology to remove the superficial layers of material and to access deeper structures might be suited for such a study.

In conclusion, we have shown that HIM allows the direct visualization of three-dimensional glomerular ultrastructure in a clinically relevant model of glomerulopathy at nanometer resolution. This technology enables a much more comprehensive and detailed characterization of glomerular architecture, including podocytes, endothelium and the interface between them. This opens up a timely opportunity to uncover and rediscover anatomic features of various glomerulopathies for disease diagnosis, differentiation and more importantly, for the understanding of the specific cellular and molecular processes associated with sophisticated morphological features of various glomerulopathies.

## Materials and Methods

### Animal experiments

All animal experiments were conducted according to the National Institutes of Health Guide for the Care and Use of Laboratory Animals and were approved by the Washington Univ. Animal Studies Committee and the Massachusetts General Hospital Institutional Committee on Research Animal Care. Adult C57BL/6 J *Col4a3* knockout mice (*Col4a3*−/− and *Col4a3*+/−) and *Cd2ap*-knockout mice were previously described^33,58^. Adult male WT mice (C57BL/6 J) were used for the lipopolysaccharide (LPS) injection experiments. LPS was purchased from Sigma-Aldrich (St. Louis, MO). WT mice were subjected to a single injection of LPS at a dose of 200 µg intraperitoneally, then sacrificed 24 hours after the LPS injection. Adult male C57BL/6 J mice were used as WT controls. All mice had free access to tap water and standard mouse chow. Mice were anesthetized with pentobarbital sodium (60 mg/kg body weight intraperitoneal injection, Nembutal, Abbott Laboratories, Abbott Park, IL) and perfused through the left cardiac ventricle at the rate of 10–15 ml/min with phosphate-buffered saline (PBS, 0.9% NaCl in 10 mM phosphate buffer, pH 7.4) for 5 min, followed by modified paraformaldehyde-lysine-periodate (PLP) fixative containing paraformaldehyde (4%), lysine (75 mM), sodium periodate (10 mM) and sucrose (150 mM) in 37.5 mM sodium phosphate at the same rate for 5 min^28^. Spot urine was collected for analysis of proteinuria at the time of sacrifice. For hematoxylin and eosin (H&E) staining, tissues were post-fixed overnight at 4 °C in modified PLP. For TEM and HIM analysis, we post-fixed the tissues overnight in 2% glutaraldehyde (GA) in 0.1 M sodium cacodylate buffer, pH 7.4 (Electron Microscopy Sciences, Hatfield, PA). Tissues were then washed with PBS and stored at 4 °C in PBS containing 0.02% NaN_3_ until processing for the critical point drying process.

### Alcohol replacement and critical point drying (CPD)

Thin (~500 μm) kidney slices were exposed to a series of graded methanol solutions with the following schedule and methanol dilutions: 25% in PBS for 60 min, 40% in PBS for 45 min, 60% in ddH_2_O for 45 min, 80% in ddH_2_O for 45 min, all at room temperature, followed by 80% in ddH_2_O overnight at 4 °C, and then 100% at room temperature for 60 min^59^. For each incubation performed at room temperature, the methanol solution was refreshed halfway through its duration. The kidney slices were then placed in metal baskets and CPD was performed using a Samdri-795 apparatus (Tousimis Research Corporation, Rockville, MD) as described previously^59^. Tissues were maintained at supercritical parameter values (>1000 psi, >42 °C) for 4–5 min. and the pressure was subsequently reduced slowly (at a rate of <100 psi/min).

### Helium ion microscopy

Helium ion microscopy (HIM) was performed using an Orion helium ion microscope (Carl Zeiss Microscopy, Peabody, MA) as previously described^28,59^ at a 35 keV beam energy with a probe current ranging from 0.1 to 1.5 pA. No conductive sample coating was performed prior to imaging. Charge control was achieved with a low energy electron flood gun. Only brightness and contrast adjustments were applied as post-processing procedures in Adobe Photoshop version 9.0.2 software (Adobe Systems, San Jose, CA).

### H&E staining and urinalysis

Fixed kidney tissues were paraffin-embedded and sectioned. 5-µm thick sections were then processed for H&E staining. For proteinuria analysis, 2 µl spot urine samples from each mouse were mixed with SDS-sample loading buffer and then underwent 10% SDS-PAGE. The gels were stained with Coomassie blue for 1 hour and washed with ddH_2_O for 1 hour. Bovine serum albumin (Santa Cruz Biotechnology, Dallas, TX) was run as a control.

### Transmission electron microscopy

Fixed kidney tissues were post-fixed in 1% osmium tetroxide in cacodylate buffer for 1 hour at room temperature, and then subjected to dehydration through a graded series of ethanol solutions up to 100%. Subsequently, they were infiltrated with Epon resin (Ted Pella, Redding, CA) in a 1:1 solution of Epon and 100% ethanol overnight on a rotator and then embedded in fresh Epon at 60 °C overnight. Using an EM UC7 ultramicrotome (Leica Microsystems, Bannockburn, IL), tissues were cut into thin sections, and then collected onto formvar-coated grids and stained with uranyl acetate and lead citrate. Sections were examined in a JEM 1011 transmission electron microscope (JEOL, Peabody, MA) at 80 kV. Images were taken by an AMT digital imaging system (Advanced Microscopy Techniques, Danvers, MA)^60^.

### Statistical analysis

Statistical analysis was performed according to the Handbook of Biological Statistics by Dr. John H. McDonald, Univ. of Delaware (http://www.biostathandbook.com/index.htm). The difference between individual groups was assessed by Student’s *t*-test, with significance set at a *P* value < 0.05. Data is expressed as mean ± standard error of the mean (SEM). Error bars represent SEM in each graph.

## Electronic supplementary material

Supplemental information
