# Supplementary material for: Ultrastructural Characterization of the Glomerulopathy in Alport Mice by Helium Ion Scanning Microscopy (HIM)
Source: Sci Rep. 2017 Sep 15;7:11696. doi: 10.1038/s41598-017-12064-5 (PMC5601433; doi:10.1038/s41598-017-12064-5)
Supplement: Supplementary file 1 — Supplemental information [file 41598_2017_12064_MOESM1_ESM.pdf]

SUPPLEMENTARY INFORMATION

for

Ultrastructural Characterization of the Glomerulopathy in Alport Mice by Helium Ion Scanning Microscopy (HIM)

Kenji Tsuji[1], Hani Suleiman[2,3], Jeffrey H. Miner[3], James M. Daley[4], Diane E. Capen[1], Teodor G. Păunescu[1] and Hua A. Jenny Lu[1,*]

1. Center for Systems Biology, Program in Membrane Biology and Division of Nephrology, Department of Medicine, Massachusetts General Hospital, and Harvard Medical School, Boston, MA, USA.
2. Department of Pathology and Immunology, Washington University School of Medicine, St. Louis, MO, USA.
3. Division of Nephrology, Washington University School of Medicine, St. Louis, MO, USA.
4. Research Laboratory of Electronics, Massachusetts Institute of Technology, Cambridge, MA, USA.

*Corresponding author: Hua A. Jenny Lu, MD, PhD, CPZN 8150, 185 Cambridge Street, Boston, MA 02114. Tel: 617 724 9815, Fax: 617 643 3182, email: halu@partners.org

Supplementary Figure

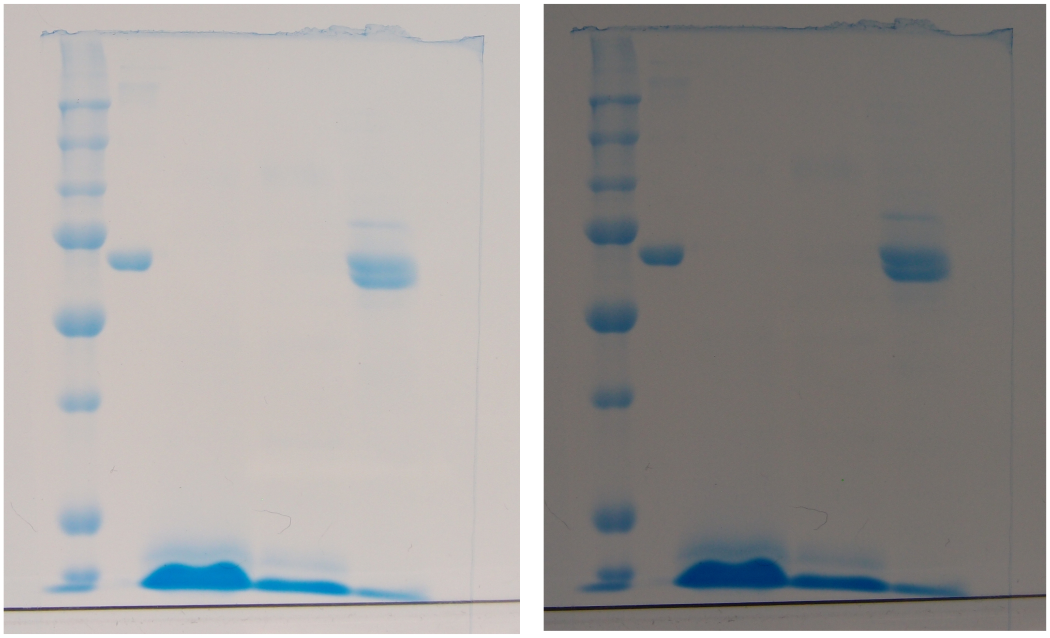

Figure S1: Analysis of proteinuria

The whole gel pictures of Figure 1b are shown.
